# Duplication of urethra: A case report from Nepal

**DOI:** 10.1002/ccr3.6925

**Published:** 2023-02-21

**Authors:** Sajina Thapa, Shila Awal, Sunil Basukala, Narayan Thapa, Bikash Bikram Thapa, Suresh Thapa

**Affiliations:** ^1^ Nepalese Army Institute of Health Sciences Kathmandu Nepal; ^2^ Department of Surgery Shree Birendra Hospital Kathmandu Nepal

**Keywords:** pre‐pubic sinus, urethral duplication

## Abstract

Urethral duplication is uncommon with few cases reported in the literature. We report a case in which a patient presented with discharge from proximal part of penis since childhood and recent history of infection. The diagnosis of pre‐pubic sinus was made and complete excision of the sinus tract was done.

## Graphical abstract

Intra operative photograph showing complete sinus, a rare entity, which when diagnosed timely and treated will lead to a better quality of life.

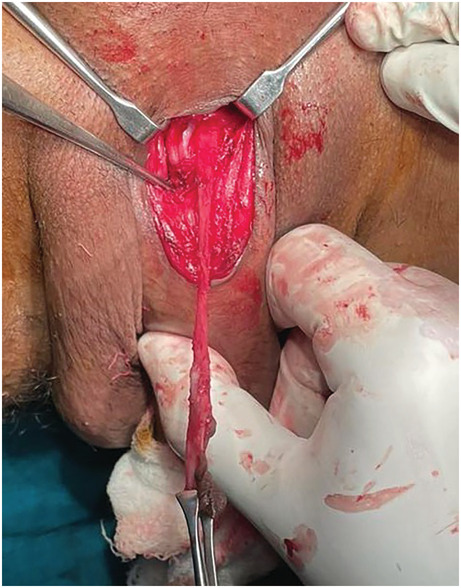

## 1 INTRODUCTION

Duplication of the urethra is a rare congenital abnormality affecting males more commonly.^1^, ^2^ Clinical manifestation depends on the variation of anatomic patterns present.^3^ Urethral duplication can either be a simple accessory blind urethral tract or an independent complete urethral tract emerging from the bladder.^4^ Duplication is more prevalent in the sagittal plane and is divided into dorsal and ventral duplication, with fewer instances of urethras lying side‐by‐side.^2^, ^5^ The horizontal duplication may present with phallus duplication or complete bladder duplication.^1^ Effmann et al composed the most followed classification of urethral duplication as this is deemed comprehensive and accurate both anatomically and clinically.^2^, ^6^ However, it failed to distinguish sagittal from coronal collateral duplication.^2^, ^7^ Numerous hypotheses have been put forward; however, there is ambiguity in regard to its embryology as the same explanation does not apply to all subdivisions of urethral duplication.^2^, ^3^ Management is based

on the type of duplication and the expression of symptoms.^2^ We report our experience with a case of urethral duplication in a male and discuss the management of this anomaly.

## 2 CASE PRESENTATION

**FIGURE 1:**
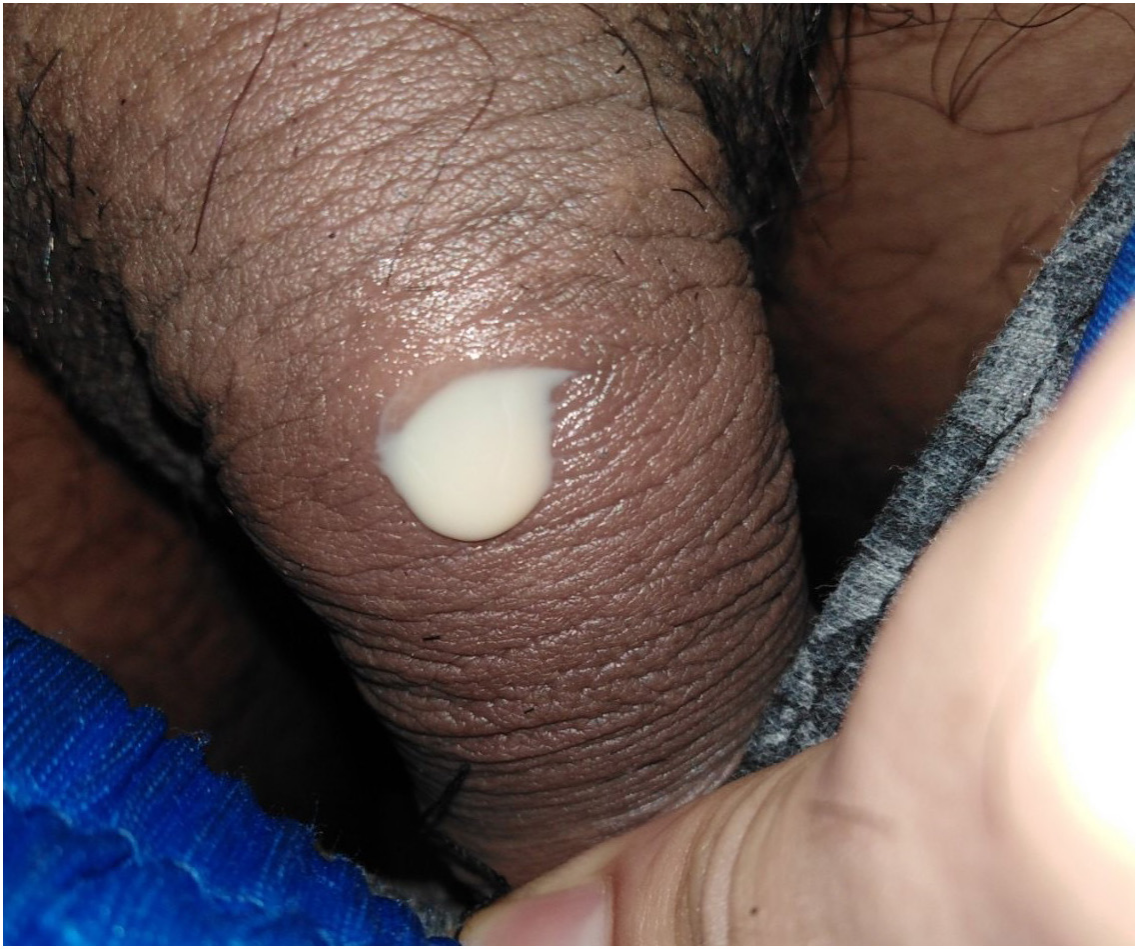
Preoperative photograph showing an accessory meatus located in the midline of the dorsal proximal aspect of the penis.

A 24‐year‐old male presented to the surgical OPD with a complaint of continuous whitish mucopurulent discharge from the dorsal aspect of the proximal part of his penis for 5 days (Figure 1). He also had dysuria and fever associated with chills and rigors. Prior to this, he had not sought any medical intervention as he was asymptomatic and had no problems except having intermittent discharge from the same site since childhood. Physical examination demonstrated an opening with discharge over the dorsal proximal part of his penis (Figure 1).

**FIGURE 2:**
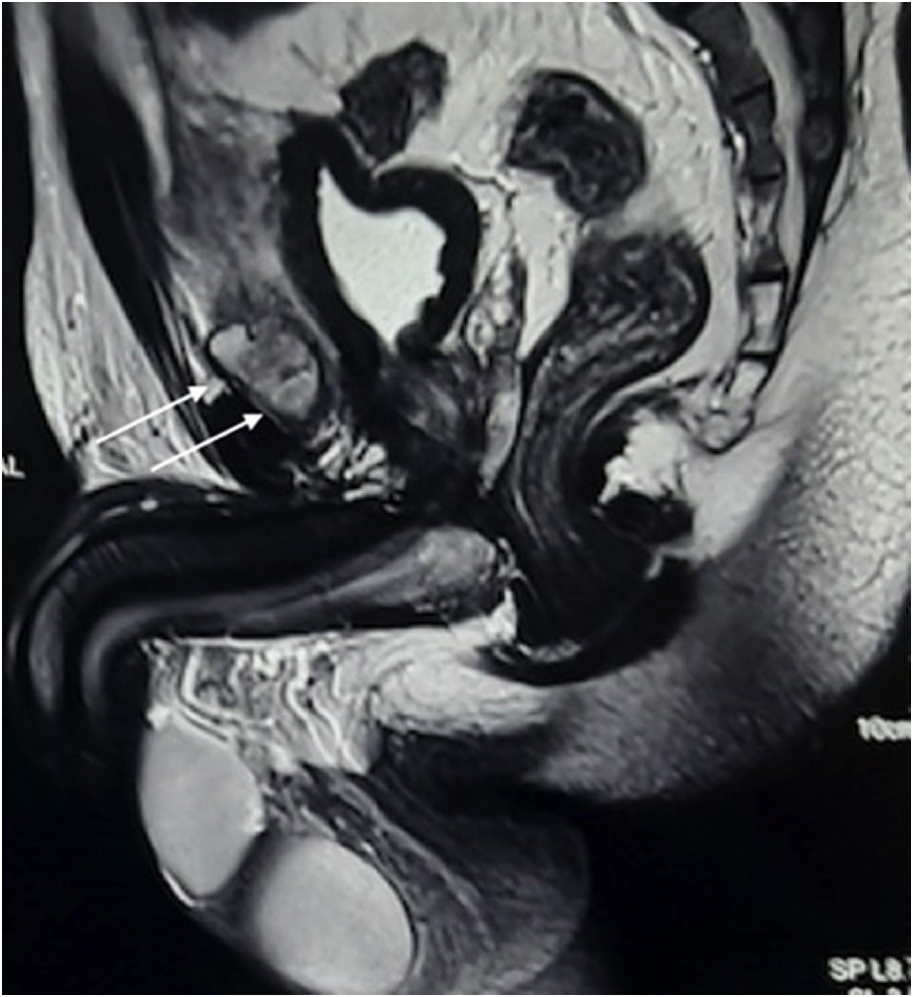
Fluid collection (white arrows) in the retropubic space of Retzius with restriction of diffusion, relation, and edema with urethral duplication.

An imaging study suggested that the opening on the dorsal proximal penis did not communicate with the bladder and normal urethra. Urgent USG revealed a collection of loculated abscesses superior to the urinary bladder. MRI scan was done which showed an enhancing thin‐walled multi‐septated loculated, high T2/STIR signal intensity fluid collection in the retropubic space of Retzius with restriction of diffusion, relation, and edema in surrounding fat, most likely an abscess and minimal altered high T2 FS signal intensity in subcutaneous plane dorsal to corpus cavernosa of the penis, likely urethral duplication (Figure 2). It is equally important to screen for other urinary tract anomalies as it can detect any undiscovered pathologies which may minimize renal damage and thereby improve the quality of life.

**FIGURE 3:**
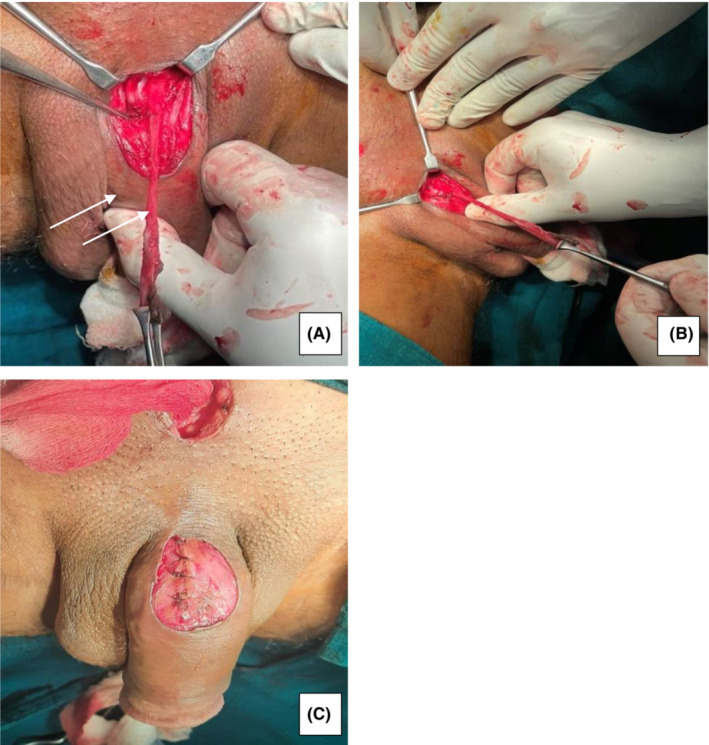
A and B Intraoperative photograph showing the complete sinus, C Postoperative photograph taken after correction of the anomaly.

Laboratory analysis revealed WBC‐13500/C.mm, Platelets‐214,000/C.mm, Neutrophil‐86%, Lymphocyte‐13%, pus culture and sensitivity—no growth, Hb‐16.4%, blood culture was sterile. Urine analysis showed trace proteins with two epithelial cells/hpf. The patient was planned for complete excision of the accessory urethra from the normal urethra with drainage of collection. Before surgery, a cystourethroscopy was performed that showed a normal urethra, a normal intact external urethral sphincter, and a normal prostatic urethra; the bladder neck, and bladder were observed and were also normal. This was followed by exploration and drainage of 20 mL pus. The skin was completely degloved, and the accessory urethra was excised down as deep as possible behind the symphysis pubis. Then, an excision of a 10 cm long urethral tract extending up to the retropubic space was done (Figure 3A–C).

Two drains were placed in the retropubic space. The post‐operative period was uneventful. The patient was managed with IV amikacin and ceftriaxone for 7 days in the surgical ward and was discharged on oral antibiotics for five more days based on the clinical improvement of the patient. Histopathological examination of the excised tissue was suggestive of the prostatic urethral tract lining with chronic inflammation. During the follow‐up, persistent discharge from the surgical site was noted following which Citrobacter freundii was isolated from the wound swab culture and sensitivity test. Exploration and debridement were done along with oral antibiotics such as ciprofloxacin and azithromycin for 5 days. The wound was allowed to heal by secondary suturing. This could have been prevented by performing an extended exploration with meticulous drainage of the collection of pus in the retropubic region. After regular dressing and follow‐up, the patient recovered well with good cosmetic and functional results. An unobstructed urinary tract that was free of infection was noted. The review ultrasonography of the pelvis after completion of the antibiotic dose showed a resolution of the size of the retropubic abscess.

## 3 DISCUSSION

Urethral duplication is a rare congenital anomaly that can present with varying presentations. This anomaly has been described mostly in males, few cases in females have been reported.^7^ Duplication usually occurs in the sagittal plane, less commonly side by side.^2^ Our case was also an instance of a male patient with urethral duplication occurring in sagittal plane. A lot of hypotheses have been proposed for urethral duplication, including ischemia, abnormal lacunar fold duplication, abnormality of Mullerian duct, and urogenital sinus.^3^ However, the embryology is unclear as described in the literature.

In males, urethral duplication is classified into three types (Effmann's classification)^4^:

TYPE I—Blind incomplete urethral duplication (accessory urethra).

1. Distal‐opens on the dorsal or ventral surface of the penis but does not communicate with the urethra or the bladder.
2. Proximal‐opens from the urethral channel and ends blindly in the periurethral tissue.

Type II—Complete patent urethral duplication.

It is further divided into two types: A (two meatuses) and B (one meatus).

Type III—Urethral duplication as a component of partial or complete caudal duplication.

Based on the classification given above, our case falls within TYPE I‐A category. Presentations depend upon the type of anatomic variability of the anomaly. Patients may present with a double stream, urinary incontinence, outflow obstruction, recurrent urinary tract infection, sometimes with sepsis; or there may not be any symptoms.^3^, ^8^ Physical examination usually reveals two meatuses. Other findings may include associated anatomical abnormalities, such as ambiguous genitalia, epispadias, hypospadias, and chordee.^6^ Our case presented with mucopurulent discharge and dysuria from dorsal aspect of his penis with an accessory meatus. Urethral tract duplication can coexist with other upper urinary tract anomalies for which thorough investigation is to be performed. In our case, there was no other anatomical defect noted. Investigations such as voiding cystourethrography, retrograde urography, urethrocystoscopy, and intravenous urography depict a clear picture of the anatomical malformation.^3^, ^8^ Patients are treated based on their presentation. Asymptomatic patients can be left untreated whereas those having bothersome symptoms should undergo surgical excision and construction as required.^8^ Since, prognosis depends on type and accompanied anomalies, urethral duplication requires individualized surgical approaches.^9^ Likewise, our patient was having fever, dysuria, and intermittent discharge, for which he underwent excision of the accessory urethra. Among various surgical procedures; excision, ligation, fulguration, and sclerosis of the accessory channel have been described well.^6^ Multistage urethral reconstruction is necessary depending upon the type of abnormality.^3^ Simple accessory duplicated urethras may be fulgurated with an electrode and allowed to heal and close. A single meatus can be created by dividing the septum between the two meatuses if both of the urethrae are functional and close to each other on the glans.^1^ A rare type IIA2 (Y type) can be treated with complete excision of the accessory urethra that opens into the scrotum from the normal urethra.^10^ This complex variety may need extensive urethroplasties requiring tissue transfer, such as buccal mucosa grafts.^1^ End‐to‐side urethrourethrostomy can be done in some cases of urethral duplication.^11^

## 4 CONCLUSION

Duplication of the urethra is often overlooked due to its rarity. Therefore, the treating medical professional need to consider the possibility of having this condition, especially if the patient is to present with the aforementioned signs and symptoms indicative of urethral duplication. This can lead to early and accurate diagnosis, hence resulting in the timely management and better prognosis of the disease. Moreover, this case, with the help of histopathological examination, suggests that prepubic sinus is a variant of dorsal urethral duplication.

## AUTHOR CONTRIBUTIONS

Sajina Thapa, Shila Awal: information of the case, wrote the manuscript, reviewed the literature, and revised the manuscript for important intellectual content. Sunil Basukala, Narayan Thapa: revised and edited the manuscript. Bikash Bikram Thapa, Sajina Thapa: responsible for the integrity of the work as a whole.

## CONFLICT OF INTEREST STATEMENT

The authors declare no conflict of interest.

## ETHICS STATEMENT

This study did not involve experiments on humans and animals.

## CONSENT

Informed and written consent was taken from the patient prior to his inclusion in this study.

## Data Availability

Data openly available in a public repository that issues datasets with DOIs.
